# Precision farming in aquaculture: assessing gill health in Atlantic salmon (*Salmo salar*) using a non-invasive, AI-driven behavioural monitoring approach in commercial farms

**DOI:** 10.1186/s44365-025-00020-8

**Published:** 2025-08-15

**Authors:** Meredith Burke, Dragana Nikolic, Pieter Fabry, Hemang Rishi, Trevor Telfer, Sonia Rey Planellas

**Affiliations:** 1 Institute of Aquaculture, Faculty of Natural Sciences, University of Stirling, Stirling, UK; 2 Observe Technologies, Richmond, UK

**Keywords:** Atlantic salmon, Aquaculture, Fish behaviour, Welfare, Gill health, Machine learning, Precision farming

## Abstract

As the aquaculture industry grows, more sophisticated technology is required to monitor farms and ensure good fish welfare, in line with the precision livestock farming concept. Using behaviour as a non-invasive monitoring tool, combined with artificial intelligence, enables greater control over farm management. This study aimed to assess temporal changes in farmed Atlantic salmon (*Salmo salar*) group behavioural profiles related to fish health and welfare. A machine vision algorithm applied to feed cameras on commercial farms was used to determine whether changes in gill health would induce visible group behavioural changes. Video cameras were deployed in all cages at two Scottish Atlantic salmon marine farms. One cage at each farm was also equipped with additional cameras (5 and 4 at sites A and B, respectively) to provide higher spatial coverage of fish behaviour and distribution. The algorithm processed video footage from these cameras and produced behavioural data termed ‘activity’ (%), which encompasses fish abundance, speed, and shoal cohesion. Additionally, gill health, Operational Welfare Indicators (OWI), mortality, and Specific Feeding Rate (SFR) were scored weekly at both sites. During summer 2023, gill health issues arose at both farms, leading to fish stress reflected in the behavioural data. For two months prior to the onset of poor gill health, the average (± standard deviation) activity levels of the fish across all cages were 25.6 ± 10.5% and 24.9 ± 7.0% for Farm A and B, respectively. After gill health was compromised, the activity rose significantly for two months in all cages with a mean of 43.6 ± 15.1% and 32.6 ± 9.6%, respectively. A generalised linear mixed model revealed that Proliferative Gill Disease (PGD) was the main driver of this increase in activity. This increase in activity coincided with fish migration to the centre of the cage, meaning tighter shoaling, which is a normal stress response often seen in relation to predators and other environmental or health stressors. The use of behaviour as a non-invasive welfare indicator and the potential to use artificial intelligence to automate the process of behavioural identification allows farmers to improve welfare conditions and ensure industry sustainability.

**Supplementary Information:**

The online version contains supplementary material available at 10.1186/s44365-025-00020-8.

## Introduction

Measuring the behaviour of cultured fish is challenging but critical for aquaculture, as it provides valuable insights into fish health and welfare being one of the 5 domains used to understand the welfare status of the animals [44]. However, observing behaviour is inherently difficult because fish are underwater, often with poor visibility, and it can be unclear which behaviours are most useful to monitor. Previous studies on the behaviour of farmed Atlantic salmon smolts (*Salmo salar*) in sea cages have focused on the environmental drivers of their swimming depth and speed [8, 53]. Salmon appear to distribute themselves heterogeneously throughout the cage, largely basing their preferred depth on environmental gradients, such as temperature and photoperiod [52, 53]. Fish shoaling, when multiple individuals group together socially, is a common anti-predator stress defence mechanism, however fish may shoal as a response to other stressors like changes in environmental or health status [20, 23, 56]. A previous study found that an unpredictable stressor (confinement) resulted in a higher shoal cohesion response in sea bass [10] and a temporal preference for higher temperatures in zebrafish as a stress coping mechanism [57]. However, there are few studies that explore the role that parasites and disease may play on group behaviour of fish in sea cage systems, with most studies considering behaviours (e.g. locomotion, foraging, shelter use) in laboratory settings (e.g. [55, 60, 62]).

Studies investigating fish response to stressors have traditionally employed invasive methods such as using plasma cortisol analysis (e.g. [35, 39, 61]) requiring crowding, handling, anaesthetising, and blood sampling which are inherently stressful for the fish. However, using behaviour as a non-invasive metric for detecting poor fish health holds promise for enhancing fish welfare [36]. Behavioural changes often precede outward signs of illness, providing farmers with an early warning signal to prevent disease rather than merely treating the symptoms [24]. Additionally, changes in feeding behaviour serve as a critical indicator of potential disease or stress in fish. Research has demonstrated a decrease in feeding motivation in response to acute stressors across vertebrates [17]. Specific feeding rate, a composite measure incorporating specific growth rate and feed conversion rate, is widely utilised by the aquaculture industry to gauge feeding efficacy. Higher specific feeding rates signify the implementation of efficient and effective feeding regimes [29]. These non-invasive tools represent examples of Operational Welfare Indicators (OWI), measures used to assess the overall welfare status of cultured fish and are pivotal for assessing fish welfare on-site within production systems and husbandry practices [51]. OWIs encompass both indirect environment-based and direct animal-based measures, which can be further categorised at a group or individual level. Implementation of OWIs is indispensable for ensuring that the welfare needs of fish are adequately addressed. Standardised tools have been developed to monitor fish welfare through direct observations, focusing on a subset of individual animals within cages for external signs of injury or illness, as well as through assessments of group-level behaviours such as feeding patterns.

Over the last decade, the increase in marine fish gill diseases is recognised as a major health and welfare problem worldwide [5]. Compromised gill function disrupts fish respiration and osmoregulation, causing breathing difficulties, and loss of salt and blood gas control. There are many causes of gill disease including parasitic gill disease, viral/bacterial gill disease, plankton/algae associated gill disease, or chemical-related gill disease [5, 58]. The parasite *Neoparamoeba perurans*, through the development of Amoebic Gill Disease (AGD) is often the most reported due to the distinctive pathology in salmon and is recognisable on-site [5]. AGD can cause typical losses to the Scottish salmon industry of 10–20%, with some sites as high as 70% [42, 63]. It is also the cause of significant mortalities in commercial salmon fisheries in Australia, USA, Canada, France and Ireland. However, gill disease is often multifactorial, due to a combination of one or more of the above causes,this is called complex gill disease (CGD; [19]). CGD encompasses Proliferative Gill Disease (PGD), which is a gill condition described in Scotland and Ireland as a generic term for the proliferative changes in the gill epithelium while causing gross lesions easily identified in the field [5]. It can result from the interaction of environmental and husbandry conditions as well as infection by pathogens and parasites (such as *N. perurans*), particularly during the summer and fall months. While AGD can be diagnosed through gill sampling and histopathology to confirm the presence of the parasite, commercial producers typically assess gill health (both AGD and PGD) on-site using a categorical scoring system [65].

The progression of climate change and increased sea temperatures is directly linked to an increase in the organisms that cause damage to the gills and water quality changes, algal blooms, handling, and temperature changes can all have a cumulative stress effect increasing susceptibility to gill disease [18, 28]. Additionally, cumulative stressors can result in susceptibility to other diseases such as infection by copepod parasites, or sea lice (*Caligus elongatus* and *Lepeophtheirus salmonis*; [19, 54]). Sea lice have both lethal and non-lethal impacts on salmon, primarily resulting in chronic stress and reduced growth rate, with *L. salmonis* reported as the largest parasitic threat to the salmon aquaculture industry [50, 64, 69, 70].

Advancements in artificial intelligence (AI) has resulted in improved monitoring techniques especially in the ocean sector where usual methods are not feasible. AI can enhance real-time or near-real time monitoring by automating the collection and analysis of data [66]. AI-based algorithms have the capability to process and analyse large datasets, image or video data through machine vision techniques. AI can detect patterns and thereby any factors contributing to anomalies to those patterns. Thus, it can contribute to the development of early-warning systems, such as the detection of environmental or health-related parameters that may cause poor welfare and mortality [41]. Moreover, they can collect high-quality data in a non-invasive manner, avoiding damage to the fish that often occurs with traditional manual methods [38]. Machine vision techniques have been used in aquaculture in the past, primarily for biomass estimation, body length measurements, fish counting and feeding behaviours [38, 68–70]. Fish behaviour in relation to stress has also been examined, however the focus is often on individual fish swimming speed, acceleration and turning angle [36]. While this type of analysis is relatively straightforward in tanks or recirculating aquaculture systems, it can be more difficult to assess in large commercial sea cages with low light, high noise, fish deformation, fish overlap and occlusion, and low field of view in larger cages [68]. The present study utilised cameras deployed within cages in two commercial aquaculture farms in Scotland, focusing on group behaviour instead. This was quantified using an AI-based algorithm developed by Observe Technologies (https://observe.tech/). The aim was to investigate fish group behaviours such as cohesive grouping or shoaling, feeding, and fish distribution in response to a common health-related stressor. This study hypothesised that changes in gill health would elicit observable group-level behavioural changes in Atlantic salmon. To test this, long-term behavioural data were collected and analysed a posteriori. The findings support the potential of precision fish farming (PFF), particularly when combined with system modelling and machine learning, as a significant advancement for aquaculture systems and a promising approach to addressing welfare-related stressors.

## Methods

### Study sites

**Fig. 1:**
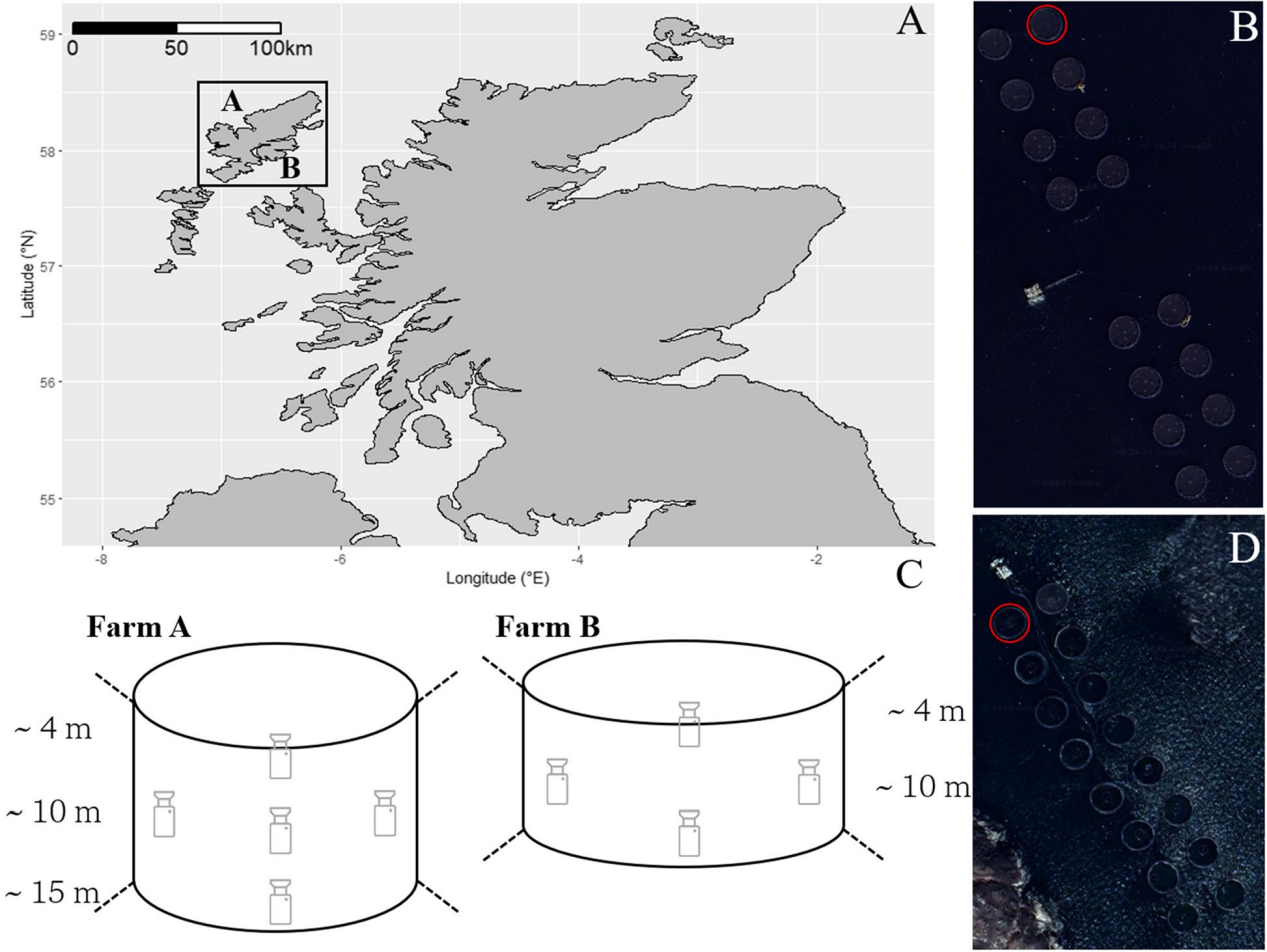
Overview of the study site. **A** Map of Scotland showing the location of the of the Atlantic salmon aquaculture farms on the Western Isles. Satellite image providing an aerial view of the Farm A (**B**) and Farm B (D; from Google Earth) with study cages outlined in red. **C** Layout illustrating the placement of cameras within the two study cages

The two commercial Atlantic salmon farms used in this study are located on the Isle of Harris and Lewis, in the northwest of Scotland (Fig. 1 A,B,D).

Farm A consists of 16 circular cages (13 stocked during the study) in two parallel rows, each 90 m in circumference and 15 m in depth with cylindrical nets (mesh size:18 mm) and Seal Pro anti-predator nets to a depth of 10 m (Table 1). Farm B contains 16 circular cages (10 stocked during the study) in two parallel rows, each 120 m in circumference and 10 m in depth (mesh size 18 mm) with Seal Pro anti-predator nets. Additionally, the fish were initially fed ad libitum and later transitioned to a twice-daily feeding schedule, which was adjusted based on their age class.

**Table 1:** Characteristics of the two aquaculture sites included in this study

Farm	Cages stocked	Cage volume (m^3^)	Initial stocking week	Mean smolt weight at stocking (g)	Mean [range] current speed (m s^−1^) *	Mean ± std fish count per cage
A	13	9 669	2023–02-13	109	0.06 [0–0.16]	37 066 ± 13 568
B	10	11 459	2023–05-08	87	0.09 [0–0.38]	83 933 ± 19 819

^*^ The current speed was measured over 90 days prior to stocking the sites

### Data acquisition

#### Fish Behaviour

There were 4–5 video camera modules (Sony model: IPCM-3516DS385-D29-AZ3611) installed by AKVA Group (www.akvagroup.com) within one, randomly allocated cage per site pointing towards the surface. These were deployed to obtain higher spatial resolution of fish behaviour and to identify optimal camera placement within the cage for capturing behavioural data from a high proportion of the population. In Farm A, they were positioned as 3 down the centre (approximately 4 m, 9 m, 14 m), and 2 at 9 m on the inner and outer areas of the cage, respectively (Fig. 1C left). As Farm B is a shallower site, only 2 cameras were placed down the centre (4 m and 9 m), with 2 at 5 m on the inner and outer areas of the cage, respectively (Fig. 1C right). The remaining cages were equipped with a single camera pointing upwards, positioned centrally at mid-depth (~ 9 m in Farm A and ~ 7 m in Farm B), to allow comparison with the higher resolution study cage. The cameras recorded at 25 frames per second, at a resolution of 1920 × 1080 pixels, and a video bit rate of 16 Mb per second. Videos were recorded continuously during daylight hours between May (June) and September (October) for Farm A (B). Daylight hours shift as summer progresses, with lowest duration in October at 12 h and peaking in June at 18 h.

Each camera contained a pressure (TE connectivity US300; accuracy of ± 0.15% full scale output) and temperature sensor (RDO PRO-X; accuracy of ± 0.1ºC and resolution of 0.01 ºC), which recorded continuously for the duration of the study. Additionally, there were single cameras deployed in the centre of the remaining cages in each farm, allowing for activity data from these cameras to be compared to the study cage to investigate whether similar trends were observed using a single-camera system.

Video recordings were analysed using an algorithm developed by by Observe Technologies (www.observe.tech), designed to extract behavioural data relevant to aquaculture operations. This algorithm employs a patented (EP3644717) convolutional neural network to heuristically estimate fish activity by identifying key behavioural features. These features include, but are not limited to, schooling cohesion, average distance from the camera, the number of fish detected per frame, and swimming speed. Rather than tracking individual fish, the model is trained globally, recognising behavioural patterns from annotated datasets. Since all features are processed within a shared network architecture until the final layer, their individual contributions to the overall activity score remain undefined. The model continuously refines its parameters through standard deep-learning optimisation techniques, generating a normalised activity score ranging from 0 to 100% (example in Fig. A.1).

To ensure reliability, the model undergoes comprehensive validation using independent training, validation, and test datasets, following best practices in deep learning (Table A.1). Further, aquaculture specialists routinely assess the model’s outputs to verify their practical relevance, and for this study, they conducted an additional review to ensure accuracy before the data was provided to university researchers. Independent validation of the algorithm was also carried out by two observers from the University of Stirling, using video footage captured by the same AKVA feed cameras. The algorithm’s'activity'output was correlated to fish abundance counts from 55 randomly chosen video stills using Pearson correlation (α = 0.05). Additionally, the Intraclass Correlation Coefficient (ICC) was calculated to assess interobserver reliability.

#### Fish health and welfare

Each farm provided health and welfare-related parameters used in this study. Daily Specific Feeding Rate (SFR) was recorded for each cage as the % body weight fed:

$$\frac{\mathrm{feedgiven}}{\mathrm{fishbiomass}}\times100$$

where biomass was determined through sample and mortality weights and number of fish in each cage.

At commercial aquaculture sites, farmers visually assess fish for OWIs, sea lice, and gill health. Given the demanding nature of farm operations and limited staffing, manual assessments remain a standard and practical protocol. On a weekly or bi-weekly basis (depending on water temperature), 10 fish per cage were examined for various Operational Welfare Indicators including fin and eye damage, skin lesions, scale loss and visual K-factor with severity scored from 0 (healthy) – 2 (severe; Table 2).

**Table 2:** Scoring system for Operational Welfare Indicators (OWIs) employed by farmers to assess fish welfare during the study

	Eye damage	Fin damage	Skin lesions	Scale Loss	K-Factor
0	Both eyes perfect	Dorsal, pectoral and caudal fins intact	Skin intact	All scales are intact, no patches of lost or damaged scales; Fish is ‘smooth’	Fish are well shaped and firm to touch; Has a ‘belly’
1	Damage to one eye (unilateral) Such as: - Rupture - Cloudiness - Bleeding - Cataract - Swelling	< half of fin remaining	1 + small wounds (< 10p coin); subcutaneous tissue intact	Small areas of scale damage or loss present, but skin is intact (i.e. no bleeding or lesions); Affected area is no bigger than 10p coin	Fish long, but not have as much depth as expected; Slightly soft to touch (esp. around the belly area)
2	Damage to both eyes	Little of fin remaining	Large (> 10p coin), severe wounds; muscle exposed	Multiple areas of scale loss or damage; Skin may look dimpled due to water ingress	Fish long and thin; head significantly oversized compared to body

Adapted from The Scottish Salmon Company (Bakkafrost Scotland) and FISHWELL handbook [51]

These 10 sampled fish are then examined for sea lice with counts per fish for gravid females (females with egg sacs attached). Gravid females were chosen as they serve as a key indicator for assessors when determining whether treatment is necessary [30]. Lastly, fish were assessed weekly for gill health using a standardised scoring protocol. Gill health was assessed by farmers using a standardised scorecard (Fig. A.2, evaluating lesions and lamellar thickening to assign AGD and PGD scores ranging from 0 (healthy to 5 (necrotic; Table 3). The distinct visual differences between AGD and PGD allow for rapid on-farm evaluation and treatment decisions, as laboratory diagnostics can take time to yield results. While somewhat subjective, this method is supported by farmer training and extensive experience.

**Table 3:** On-site gill health scoring system used by farmers to estimate the severity of AGD and PGD

Score		AGD	PGD
0	Healthy	No sign of infection	Healthy, no thickening
1	Very light	1 white spot/light scarring	Very slight thickening/few lamellae affected
2	Light	2–3 spots/small mucus patch	Frequent thickening but tips only
3	Moderate	Established thickened mucus patch/spot groupings up to 20% of gill	Almost all lamellae have thickened tips/some have thickenings progressing to ~ 50% of the length of lamellae
4	Advanced	Established lesions covering up to 50% of gill	Most lamellae have thickening progressing to > 50% of the length of lamellae
5	Heavy	Extensive lesions covering most of gill surface (> 50%)	Almost all lamellae are thickened along entire length

AGD details adapted from [65], PGD details adapted from The Scottish Salmon Company (Bakkafrost Scotland)

### Data collection

This study was conducted for 4 months for each site, from May 10 to September 10, 2023 for Farm A and June 22 to October 13, 2023 for Farm B, these differences in time frames were related to the later stocking of Farm B while maximising data collection. At the beginning of July for Farm A and in mid-August for Farm B, there was an increase in the activity of the fish, prompting a divide in the dataset for 2 months pre/post rise in activity. Activity data was preprocessed through a rolling average of 15 min to identify trends and remove excess noise. Additionally, activity data collected during feeding periods were excluded to ensure that it did not influence the observed behavioural patterns. Utilising the depth sensors integrated into each camera, the depth of maximum activity was determined by identifying the camera with the highest activity at each time point and recording its corresponding depth.

### Statistical analysis

All statistical analyses were conducted using the Python programming language (https://www.python.org/), with statistical significance set at α = 0.05. To independently validate the algorithm, 55 videos were randomly selected, and the number of fish visible on screen was counted by two evaluators. These abundance counts were then correlated with the corresponding activity (%) measured at each time point. The Intraclass Correlation Coefficient (ICC) was used to assess inter-rater reliability and to determine both absolute agreement and consistency between the evaluators. Mann–Whitney test was used to determine whether there were significant differences in activity, AGD, PGD, SFR, and mortality between the two time periods for each farm. The maximum activity was used for the multi-camera study cage, and this was aggregated at 15-min intervals. Following this, model II linear regressions were performed to determine correlation between weekly-averaged maximum activity and gill health score with ANCOVA to determine significance for each farm independently. Coefficients of variation (COV) were used to determine the extent to which the level of dispersion of the depth of maximum activity was around the mean. Additionally, Aligned Ranked Transform ANOVA allowed for a comparison between each of the cages before and after the increase in activity. Lastly, a Generalised Linear Mixed Model was used to determine the effects of weekly-average temperature, AGD, PGD, and sea lice (*L. salmonis* gravid females) on weekly-averaged activity with random effects included for cages and farms. All variables were z-transformed prior to analysis. Raw data is available through DataSTORRE (http://hdl.handle.net/11667/241).

## Results

### Model validation

To validate model efficacy, the algorithm was independently validated by using videos recorded using the same AKVA feed cameras. Evaluators assessed the algorithm’s 'activity' output and demonstrated a strong correlation with fish abundance counts from 55 clips (R^2^ = 0.70, *p* < 0.001).

Independent validation demonstrated a strong correlation (R^2^ = 0.70, *p* < 0.001) between fish abundance observed in feeding camera footage by human assessors and the still images from the same videos used to calculate activity via the algorithm. The ICC determined high correlation both in the absolute agreement (ICC = 0.99) and the consistency (ICC = 0.98) between the two evaluators. Both reflect strong agreement between the observers that was statistically significant (*p* < 0.001). While fish speed and cohesion could not be separately validated, it is assumed that the 30% variation not captured is related to the speed and cohesion components.

### Farm A: activity and health

**Fig. 2:**
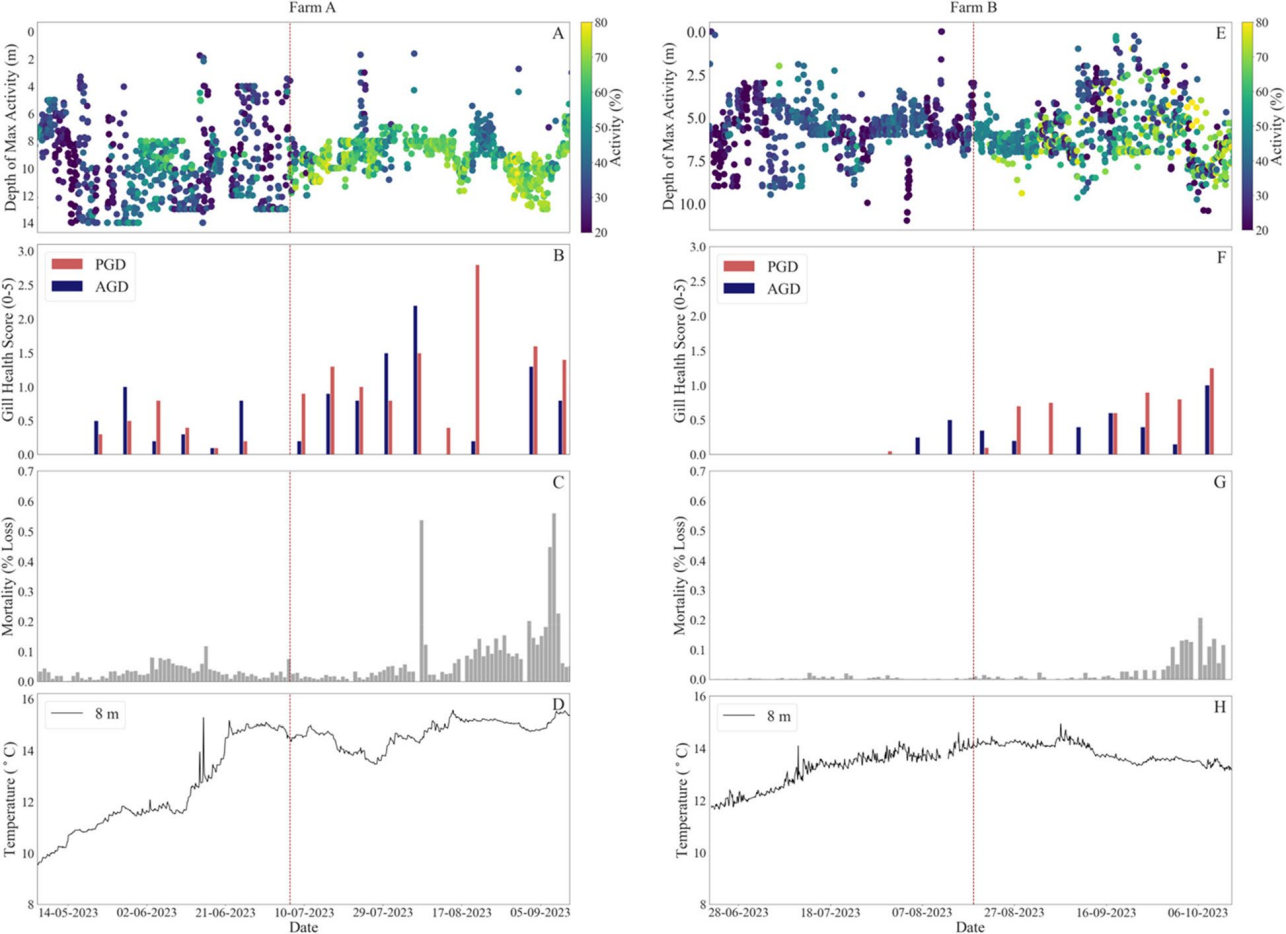
Comparative figure for Farm A (left) an Farm B (Right) with: depth of maximum activity where colours indicate the hourly-averaged amount of activity present at that depth (**A**,**E**), weekly gill health scores (0 = healthy gills, 5 = necrotic tissue), where red indicates Proliferative Gill Disease (PGD) and blue indicates Amoebic Gill Disease (AGD; **B**,**F**), daily mortality as a percent loss within the cage (**C**,**G**), and hourly temperature at 8 m depth (**D**,**H**)

In Farm A, there were seasonal effects on temperature. At the start of the study, on May 10, the temperature was 10.0 ºC within the study cage, with an upward trend reaching a peak of 14.7 ºC by June 23 (Fig. 2). Throughout the remainder of the trial period the temperature maintained a consistent range, oscillating between 14.6 ºC and 15.3 ºC, ultimately settling at 15.4 ºC by the end of the study. While only temperature data was presented in the results for both farms, oxygen, turbidity, and salinity were also monitored throughout the study period, however these parameters showed only minor daily fluctuations and did not exhibit significant trends over the sampling period. As the behavioural responses were observed over several months, daily variations in these parameters were unlikely to significantly influence long-term behavioural patterns and therefore these results are included in the appendix (Fig. A.3).

The depth of maximum fish activity, determined through the in-camera depth sensors, elucidated both the predominant depth distribution of the fish within the cage and the degree of their spatial cohesion (Fig. 2).

There was a pronounced shift in the activity and depth of the fish on July 7. In the 2 months prior to July 7, the average depth at which fish congregated was 10.0 ± 2.8 m, with a COV of 34.5%. For 2 months after July 7, this depth of maximum activity then decreased to 9.0 ± 1.4 m (U = 1.32 $\bullet$ 10^7^, *p* < 0.05) with a notable decrease in the COV to 18.7% as the fish coalesced towards the central region of the cage, indicative of heightened cohesion among individuals. This transition occurred rapidly, with the COV decreasing from 28.5% one week prior to July 7 to 13.1% the week after.

**Fig. 3:**
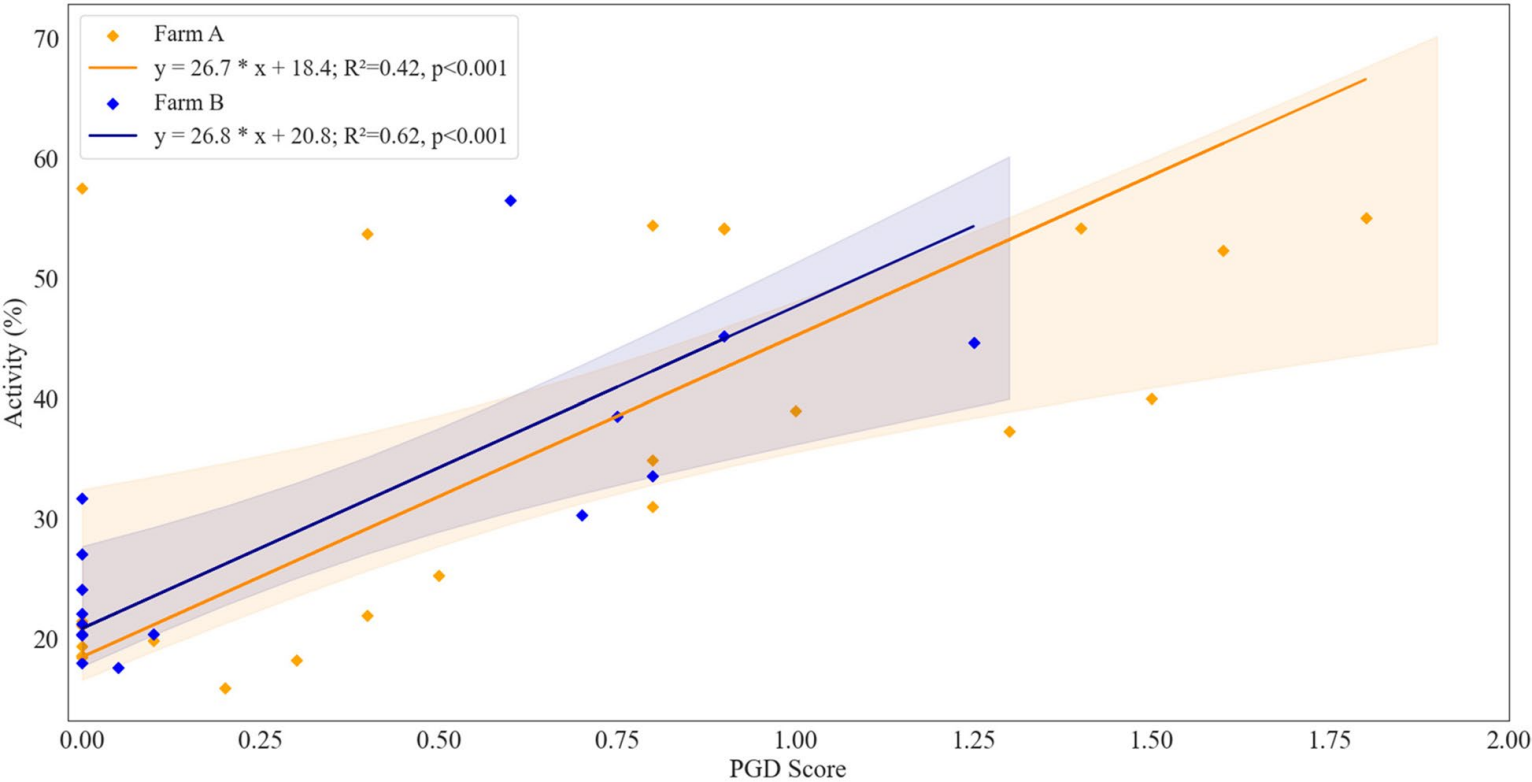
The model II linear regression for weekly activity and PGD scores for Farms A (orange) and B (blue) with confidence intervals (shaded region)

Moreover, there was a significant increase in maximum activity from 37.5 ± 13.1% to 60.8 ± 11.5% (U = 2.5 $\bullet$ 10^6^, *p* < 0.001) for 2 months before and after July 7, reflecting the intensified group cohesive behaviour as potential shoaling behaviour. This transition was rapid as well, from 32.3 ± 11.2% one week prior to 56.8 ± 17.0% on average one week post July 7. Concurrently, gill health assessments were conducted throughout this period, with the average AGD and PGD scores initially recorded as 0.4 ± 0.3 and 0.4 ± 0.2, respectively. Following the increase in activity, both AGD and PGD scores rose to 0.8 ± 0.7 and 1.2 ± 0.7 respectively. Subsequently, a correlation analysis revealed a non-significant association between AGD and activity (*p* > 0.05, R^2^ = 0.07), while a significant correlation was observed between PGD and activity (*p* < 0.001, R^2^ = 0.42; Fig. 3).

There was a significant decline in the specific feeding rate (SFR) at Farm A, from an average of 2.3 ± 0.7 prior to the augmented activity levels of the fish, to an average of 1.1 ± 0.3 after (U = 1477, *p* < 0.001). Furthermore, mortality rates within the cage exhibited a slight, though non-significant, increase from 0.03 ± 0.02% to 0.08 ± 0.1% with the increased activity on July 7 (U = 23, *p* = 0.24).

### Farm B: activity and health

The study period for Farm B commenced later in the year, on June 22, with temperatures recorded at 11.8 ºC. Over the course of the study, temperatures steadily increased, reaching 14.7 ºC by September 7, before declining slightly to 13.3 ºC by October 13.

On August 19, there was a significant increase in maximum fish activity from 32.5 ± 10.5% to 51.1 ± 19.0% (U = 5.9 $\bullet$ 10^5^, *p* < 0.001). This occurred concurrently to a significant increase in depth from 5.8 ± 1.8 m to 6.0 ± 1.9 m (U = 1.16 $\bullet$ 10^6^, *p* < 0.001), though it should be noted that this cage had a shallower depth overall. In a similar trend as Farm A, the maximum activity and COV transition quickly for the week prior and post-August 19, whereby the activity increased from 27.8 ± 10.2% to 48.7 ± 9.2%, and the COV declined sharply from 30.4% to 13.2%.

In contrast to Farm A, Farm B exhibited overall lower gill health scores, with AGD scores recorded at 0.1 ± 0.2 prior to the heightened fish activity and increasing to 0.4 ± 0.3 after. Similarly, PGD scores were recorded at 0.01 ± 0.02 prior and 0.7 ± 0.4 post-increase in activity. A significant correlation was established between AGD and activity at this site (*p* < 0.05, R^2^ = 0.35), however a stronger correlation was observed between PGD and activity (*p* < 0.001, R^2^ = 0.62; Fig. 3).

Despite a slight decrease in SFR from 1.4 ± 0.6 to 1.3 ± 0.4, this change was not statistically significant (U = 1568, *p* > 0.05). However, there was a significant increase in mortality rates, rising from 0.03 ± 0.01% to 0.2 ± 0.3% concurrent with the heightened activity observed on August 18 (U = 7, *p* < 0.01).

### Farm-wide effects

**Fig. 4:**
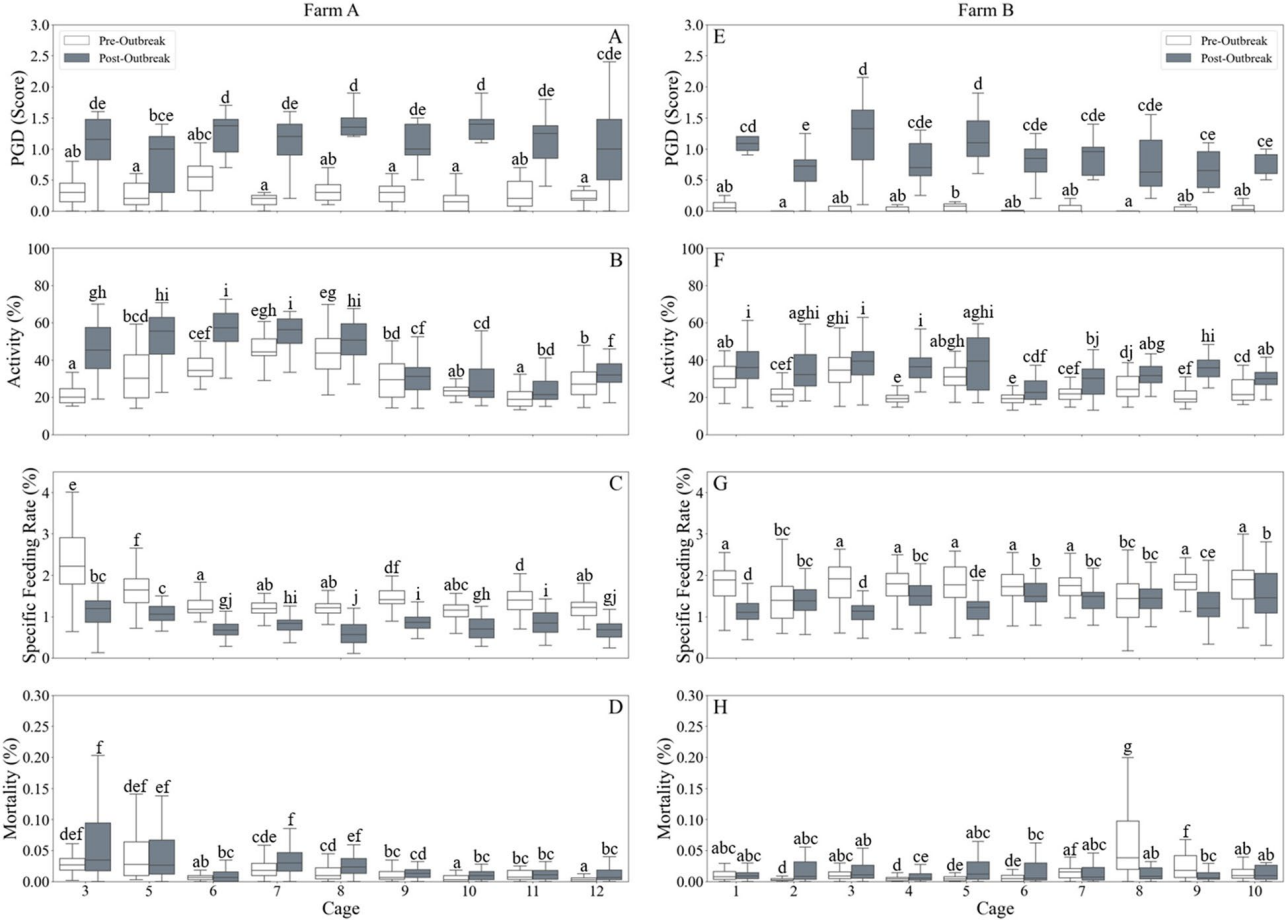
The health data from each cage in Farm A (left) and Farm B (right) including: The weekly PGD scores (**A**. **E**), the daily activity of the fish (**B**, **F**), the daily specific feeding rate (SFR; **C**, **G**), and the daily mortality (**D**, **H**). The colours indicate average 2 months pre- (white) and post- (grey) increase in activity (July 7 [Farm A]; August 18 [Farm B]). Different letters denote significant differences within and among cages per farm

The investigation extended to the remaining cages at each site, each equipped with a single camera centrally positioned at mid-depth, around 9 m for Farm A and 7 m for Farm B. A similar pattern emerged, characterised by significant increases in activity concomitant with increased PGD scores, decreased SFR, and increased mortality rates (Fig. 4).

In Farm A, 2 months prior to July 7, the activity levels were 25.6 ± 10.5%, increasing to 43.6 ± 15.1% 2 months thereafter, with all cages demonstrating a significant elevation ([3.8–22.9%; min – max difference within cages]; *p* < 0.01). Concurrently, AGD scores increased from 0.3 ± 0.06 to 0.51 ± 0.06 [0.04–0.4], with PGD scores exhibiting a more pronounced increase from 0.3 ± 0.09 to 1.1 ± 0.2 [0.4–1.2] across all cages, each showing a significant rise (*p* < 0.05). SFR exhibited a significant decline in all cages, from 1.3 ± 0.1 to 0.7 ± 0.1 ([−0.4– −1.2]; *p* < 0.05). Furthermore, there was a slight rise in mortality after July 7, on average from 0.2 ± 0.3% to 0.3 ± 0.3%, though this relatively small difference can be attributed to the low median mortality rate, with the upper quartiles in most cages indicating a trend towards higher mortality in the 2 months following July 7. There were 5 cages (6, 7, 8, 10 and 12) that did exhibit significantly elevated mortality (*p* < 0.05).

Similarly, in Farm B, the average activity levels increased from 24.9 ± 7.0% to 32.6 ± 9.6% on August 18, with all cages, except cages 3 and 5, exhibiting significant increases ([5.2–16.8%; min – max difference within cages]; *p* < 0.05). It should be noted however that the latter two cages had trends towards an increase even though it was not statistically significant. AGD scores increased from an average of 0.1 ± 0.2 to 0.6 ± 0.4 [0.3–0.7], while PGD scores increased from 0.1 ± 0.2 to 0.9 ± 0.5 which was a significant increase in all cages ([0.6–1.2], *p* < 0.05). SFR decreased from average of 1.7 ± 0.3 to 1.4 ± 0.3 [−0.06 – −0.7]; all cages were significantly lower except for cages 2 and 8 (*p* < 0.05). The average mortality rate rose similarly to Farm A, from 0.2 ± 0.9% to 0.3 ± 0.5%, again with around half the cages (2, 4, 6, 8 and 9) showing significant increases in mortality (*p* < 0.05).

Furthermore, a generalised linear mixed model was employed to examine the influence of temperature, AGD, PGD and sea lice (gravid *L. salmonis*) on activity levels. The model revealed a significant effect of PGD on activity (0.32 [0.16–0.48], *p* < 0.001), while AGD, temperature and gravid female *L. salmonis* were non-significant (*p* > 0.05). The model’s predictive capacity for activity was substantial with an R^2^_marginal_ of 0.25 [0.12—0.39; 95% confidence interval], particularly when accounting for the variation at the cage and farm levels (R^2^_conditional_: 0.60 [0.54—0.64]).

Regarding the Operational Welfare Indicators assessed at each farm, no significant increases were observed for any parameter at either farm (*p* > 0.05). Nonetheless, at Farm A, a trend toward leaner fish was discernible, evidenced by an increase in the occurrence of visual K-factor scores above 0 following the increase in activity, rising from 8 to 18 instances across all cages, though this was not mirrored at Farm B.

## Discussion

Gill disorders are a growing economic and welfare concern for the salmon aquaculture industry worldwide. Deterioration in gill health can precipitate significant mortality events, hinder salmon growth and production, and compromise overall welfare [59]. The escalating temperatures due to climate change exacerbate factors contributing to compromised gill health. These factors may encompass physical stressors, including abrasions of the gill surface, as well as pathogenic or parasitic agents such as *N. perurans*, causing AGD, hydrozoan jellyfish or other gelatinous zooplankton, as well as phytoplankton such as diatoms, which can instigate gill lesions [3, 26].

The advancement of aquaculture monitoring has been significantly enhanced with the emergence of artificial intelligence (AI). The use of AI algorithms, through machine learning methodologies, facilitates the processing of extensive datasets, including the analysis of image and video data to discern patterns and trends, thereby enabling investigations such as the present study [41]. Leveraging AI for pattern recognition can result in the detection of any anomalous behaviours, indicative of stress and compromised health responses. In this study, an algorithm based on machine vision was employed to analyse videos sourced from commercial fish farms to quantify fish group behaviour and compare temporal behavioural profiles. The resulting ‘activity’ parameter is a combination of the relative abundance of fish within the video frame, perceived average inter-fish distances, and group schooling behaviour in one metric to describe the fish behaviour overall. Since the machine vision model is trained end to end, there is no overarching weighted model that dynamically adjusting feature importance. Instead, the model learns each feature independently while estimating relative activity. This innovative approach allows researchers, as well as farmers, to establish correlations between behaviours, specifically related to group social cohesion and fish abundance in certain cage locations, and external factors, a task that traditionally demands considerable time and effort. Moreover, while many algorithms focus on individual fish behaviour [38], this approach allows farmers to observe the behaviour of the fish as a group, giving a more holistic view.

Deteriorated gill health was observed at two study sites situated within the Western Isles of Scotland. The initial occurrence was documented at Farm A in early June followed by a subsequent manifestation at Farm B in mid-August, 2023. Both sites exhibited increased levels of fish activity coinciding with higher gill health scores, particularly in terms of proliferative gill disease (PGD) scores, confirming the hypothesis that compromised gills would alter salmon behaviour. However, intriguingly, no significant correlations were discerned between the prevalence of Amoebic Gill Disease (AGD) specifically, ambient temperature or the presence of gravid female *L. salmonis.* The utilisation of gravid females in this case was to investigate whether activity could be used as an indicator for sea lice infestation, to inform managers and potentially allow for pre-emptive actions prior to visual inspections, as gravid females have been correlated to the external infection pressure [31]. However, the investigation failed to establish any significant correlation between the presence of gravid female lice and alterations in fish activity levels. It is important to note that there were few sea lice counted during the study period, as such, this may be of interest to explore further in future studies.

Most studies focus on AGD as it can be detected relatively easily through histopathology and narrowed down to a singular amoeba rather than PGD which can be caused by a variety of infectious agents and has limited diagnostic test to detect it [5]. Thus, the tests and treatments used for PGD are simply those used to test and treat AGD, specifically total histopathology score by Mitchell et al., [47], or by gross gill observations described here. This study provides further information on how PGD can impact the behaviour and welfare of the fish, that cannot be observed through AGD tests alone. It should be noted that this study does not aim to diagnose the specific causes of PGD. Although turbidity remained similar throughout the study, few micro-jellyfish were observed and no changes in plankton counts, other potential factors influencing fish behaviour cannot be ruled out. Although a more detailed diagnostic approach could provide clarity on the etiology, such methods were beyond the scope of this farm-based study, where visual gill scoring by experienced farmers is the standard practice. This method has shown moderate to good agreement with histopathological diagnosis [1], though reliability decreases when symptoms are mild [11]. Refinements to reduce subjectivity and explore additional behavioural indicators, particularly in early or mild cases, could enhance diagnostic accuracy. Despite this limitation, the observable deterioration of gills was linked to behavioural changes occurring over several months, indicating a strong relationship between gill health and fish behaviour. The authors acknowledge this as a limitation and suggest that future research incorporating more precise diagnostic techniques could further elucidate the relationship between specific gill diseases and behavioural responses.

Several OWIs were investigated in this study. These are important for fish welfare and can be easily implemented by farmers in their daily husbandry practices [51]. Various types of welfare indicators exist, each with its own set of strengths and weaknesses. For instance, environment-based indicators such as temperature and oxygen levels are rendered ineffective in the absence of extremes, as observed in this study where such indicators remained relatively stable. Consequently, animal-based indicators, both at the group- (behaviour, feeding) and individual- (gill status, external inspections of the fish, k-factor) levels, were employed. As behaviour began to change and cohesive group behaviour or shoaling was more prominent, there was a rise in gill health scores. However, there was no significant change in the outward signs of illness (e.g. no change to the eyes, fins, skin). Despite the advantage of individual animal-based indicators (ABI) in detecting outward signs of poor health, the sampling of only 10 fish per cage out of a population exceeding 30,000 individuals highlights a weakness. Additionally, relying solely on individual indicators may be insufficient, as by the time outward signs of illness become apparent, the fish may already be compromised. In contrast, group-based indicators (GBI) offer a more holistic approach, allowing for the observation of most fish within the cages. Moreover, behaviour is the only welfare indicator that provides farmers direct insight into the subjective experience of fish, while OWIs (i.e. physiological or external parameters) often reflect indirect, consequential effects of environmental or health stressors [51] and are usually based on negative experiences. Though these indirect indicators remain valuable for comprehensive welfare assessments, behaviour uniquely captures the immediate responses of fish, making it a critical tool for early detection of welfare concerns in farm settings. This means that managers can have a better understanding of the state of the fish in a non-invasive way, simply through their position in the water column and proximity to each other [43, 51].

Changes to fish behaviour can be a warning sign that the fish are experiencing stress and reduced welfare. For example, social cohesion, such as schooling or shoaling, serves as an effective antipredator adaptation in fish [21, 33, 48]. Studies found higher shoaling in response to predator stimuli in banded killifish, minnows, and zebrafish, [20, 32, 40, 46]. This response allows for the potential dilution effect, as it decreases the odds of being predated upon. This mechanism may also translate into a response to compromised health, as stressed fish are more likely to shoal [27]. Moreover, a recent study on the effect of fish schooling on swimming noise production suggested that schooling fish emit lower sound levels, potentially shielding the group from predators while optimising hydrodynamic efficiency [71]. However, the consequences of this behaviour are not fully understood,while increased shoaling is typically associated with elevated risk of disease transmission, such as AGD, among nearby conspecifics, it may also confer protective benefits through mechanisms akin to herd immunity or the dilution effect [45]. Further research is needed to clarify whether shoaling primarily facilitates the spread of pathogens or may serve as a defence strategy against them. Moreover, such aggregation behaviour can lead to overcrowding in localised areas of the cage, potentially reducing oxygen availability and further impacting welfare, although this was not observed in the present study.

Acute stressors also disrupt feeding behaviour, leading to reduced food intake and growth, which can affect their energetic costs and ultimately impact their welfare [49]. Studies have shown that all vertebrates exhibit stress-related suppression of appetite and food intake, as well as enhanced energy mobilisation [9, 22]. This phenomenon is evolutionarily advantageous, as appetite suppression reduces the risk of predation, given that foraging in the presence of predators heightens mortality rates [9]. Moreover, metabolic energy is directed toward vital activities such as respiration, locomotion, and tissue repair, all of which contribute to individual survival, prioritising these functions overgrowth and reproduction [15, 67]. This study observed a decline in specific feeding rates following the onset of gill damage. The impact of compromised gill health on feeding behaviour was demonstrated primarily at Farm A, where a significant decline in the SFR was observed following the onset of deteriorated gill health concomitant with increased activity levels. While a downward trend in SFR was also discernable at Farm B, it was not statistically significant. Reduced feeding often considered a primary symptom of stress in fish, alongside changes to swimming behaviour, underscores the importance of capturing these changes through metrics such as SFR [12].

This study also documented rises in mortality within the majority of cages at Farm A, along with discernible trends towards increased mortality, albeit not statistically significant, at Farm B. While mortality is a commonly used group-based OWI in production systems, it is important to acknowledge the inherent limitation, as it signifies an irreversible stage in the deterioration of fish welfare, precluding the implementation of corrective measures [16, 51]. The limitation is underscored by the findings in this study, as significant mortalities were observed at Farm A, correlating with the highest levels of AGD and PGD, alongside significant increases in activity. Conversely, Farm B, characterised by lesser but still notable increases in gill health issues and activity, did not exhibit significant rises in mortality in most of the cages. This discrepancy shows that relying on mortality alone as an indicator would overlook the manifestations of poor welfare discerned in this study through behavioural changes. If mortality is to remain an operational welfare indicator, it signals issues at too late a stage. To enhance its utility, individual-based assessments should be complemented by comprehensive behavioural monitoring. This combined approach enables earlier detection of welfare concerns, improving fish health and welfare outcomes.

The effect sizes differed notably between the two sites, with Farm A exhibiting poorer gill health, heightened activity levels and mortality, and more pronounced reduction in SFR compared to Farm B. The discrepancy in gill health scores between the two farms likely underlies the variation in response, with Farm A recording higher scores, resulting in a lesser increase in activity, and absence of a significant decrease in SFR at Farm B. Several factors may contribute to the observed disparities between the two sites. Firstly, it is pertinent to acknowledge the highly contextual natures of PGD, with the specific pathogenic composition underlying PGD manifestation varying across sites [34]. Furthermore, differences in environmental exposure levels warrant consideration, particularly regarding the location of Farm B, which is characterised by robust tidal-driven currents, potentially facilitating the transport of harmful agents such as *N. perurans*, micro-jellyfish, plankton, and other debris capable of damaging the gills. Additionally, temporal discrepancies in fish stocking, with Farm A stocked 3 months prior to Farm B, result in differences in fish size between the two cohorts. Lastly, variations in broodstock between the sites may further contribute to disparities in response to gill health challenges. Previous studies have elucidated the influence of genetic background on behavioural traits and disease susceptibility in Atlantic salmon [7, 14]. However, it is important to note that despite the comparatively smaller magnitude of change in activity observed at Farm B, both sites exhibited analogous behavioural shifts in response to compromised gill health. This suggests a conserved behavioural response related to survival among Atlantic salmon, irrespective of genetics, age, or environmental conditions, underscoring the impact of poor gill health-induced stress on fish behaviour.

As climate change progresses and temperatures continue to rise, the agents causing PGD are expected to proliferate, leading to higher rates of illness. Over the past 4 decades, sea surface temperature around the UK have exhibited a warming trend of approximately 0.3 ℃ per decade [13]. This warming trend may broaden the reproductive periods and improve winter survival of jellyfish and plankton, causative agents of PGD, at mid-latitudes [6, 25, 37]. Moreover, research indicates that temperatures exceeding 12 ℃ can trigger outbreaks of AGD, potentially increasing the attachment and growth capacity of *N. perurans* under elevated temperature conditions [4]. Consequently, the establishment of efficient early detection mechanisms for illnesses is imperative to mitigate stress and mortality among farmed fish [2]. This study provides insight into the role of behavior in detecting PGD, facilitating the implementation of preventative or treatment interventions.

## Conclusion

In this study, a pronounced increase in fish activity occurred at the emergence of poor gill health in two Atlantic salmon farms in Scotland. During the summer and early autumn of 2023, both farms experienced a significant increase in fish activity concomitant with the onset of PGD. This was accompanied by a decline in SFR and increase in mortality rates, particularly evident in Farm A. Given the escalating temperatures associated with climate change, it is conceivable that the prevalence of agents precipitating PGD will escalate, underscoring the need for the aquaculture industry to devise effective mitigation strategies. Gill health is a leading cause of mortality in the global Atlantic salmon industry, thus is it is imperative that studies continue in this area to improve the welfare of farmed fish. The findings of this study hold significant implications for future studies, which can delve deeper into elucidating the activity thresholds indicative of impending gill health deterioration. This could lead to the development of a non-invasive early warning system for identifying compromised gill health. Such a tool could prove invaluable in proactively addressing welfare concerns in commercial aquaculture systems.

The main challenges currently facing aquaculture are based on the improvement of best management practices and the advancement of monitoring systems, both for the aquatic environment and for assessing fish behaviour, health, and welfare. Implementing a systematic and integrated welfare assessment framework that spans the entire production cycle up to slaughter is essential to improve the understanding of basic needs of the fish, mitigating stressors and stress responses, and ultimately enhancing productivity and product quality. The development of Precision Fish Farming (PFF), incorporating system modelling and machine learning, represents a significant advancement in aquaculture technology. This approach offers promising solutions to welfare-related challenges, including those explored in the present study.

## Supplementary Information

Supplementary Material 1.

## Data Availability

The datasets supporting the conclusions of this article are available in the DataSTORRE repository, http://hdl.handle.net/11667/241.
