# Supplementary material for: Precision farming in aquaculture: assessing gill health in Atlantic salmon (Salmo salar) using a non-invasive, AI-driven behavioural monitoring approach in commercial farms
Source: Aquac Sci Manag. 2025 Aug 15;2(1):15. doi: 10.1186/s44365-025-00020-8 (PMC12369403; doi:10.1186/s44365-025-00020-8)
Supplement: Supplementary file 1 — Supplementary Material 1. [file 44365_2025_20_MOESM1_ESM.docx]

**Appendix A**

*Figures:*


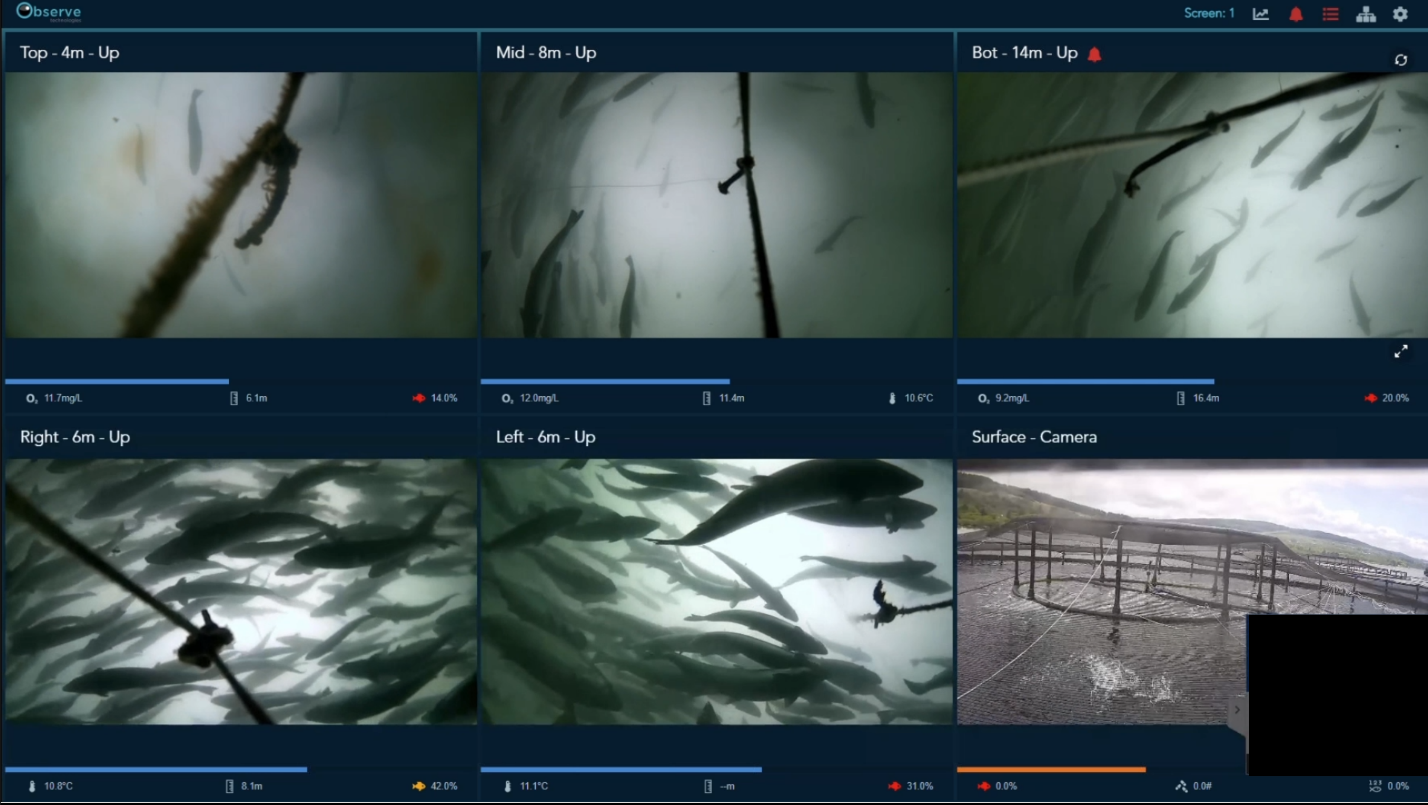


Figure A.1 Example of a screenshot from a video showing the five cameras at Farm A. Each panel displays temperature, depth, and oxygen (when available), with the coloured fish icon in the bottom right indicates the calculated activity (%) at that moment.


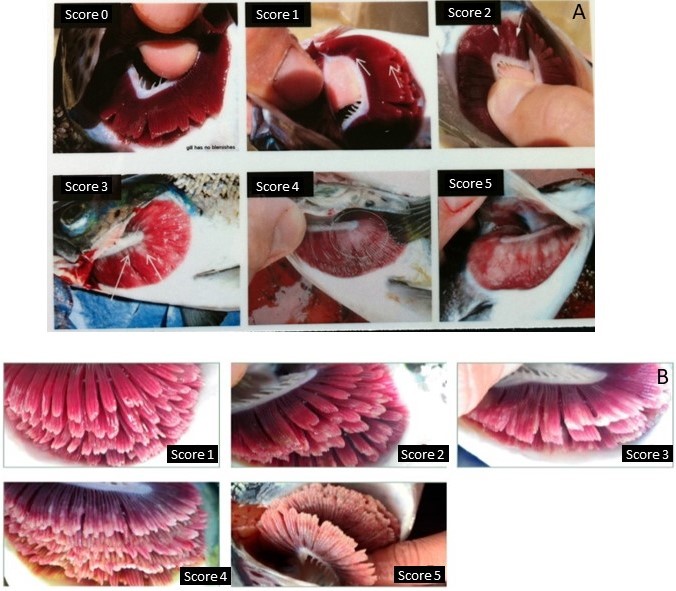


Figure A.2 The scorecards used by farmers to assess AGD (A) and PGD (B) scores on-site. Images courtesy of Bakkafrost Scotland, with contributions from Skretting (Mark Adams and Vet-Aqua International; A) and Marine Harvest Scotland (now Mowi Scotland; B).

*
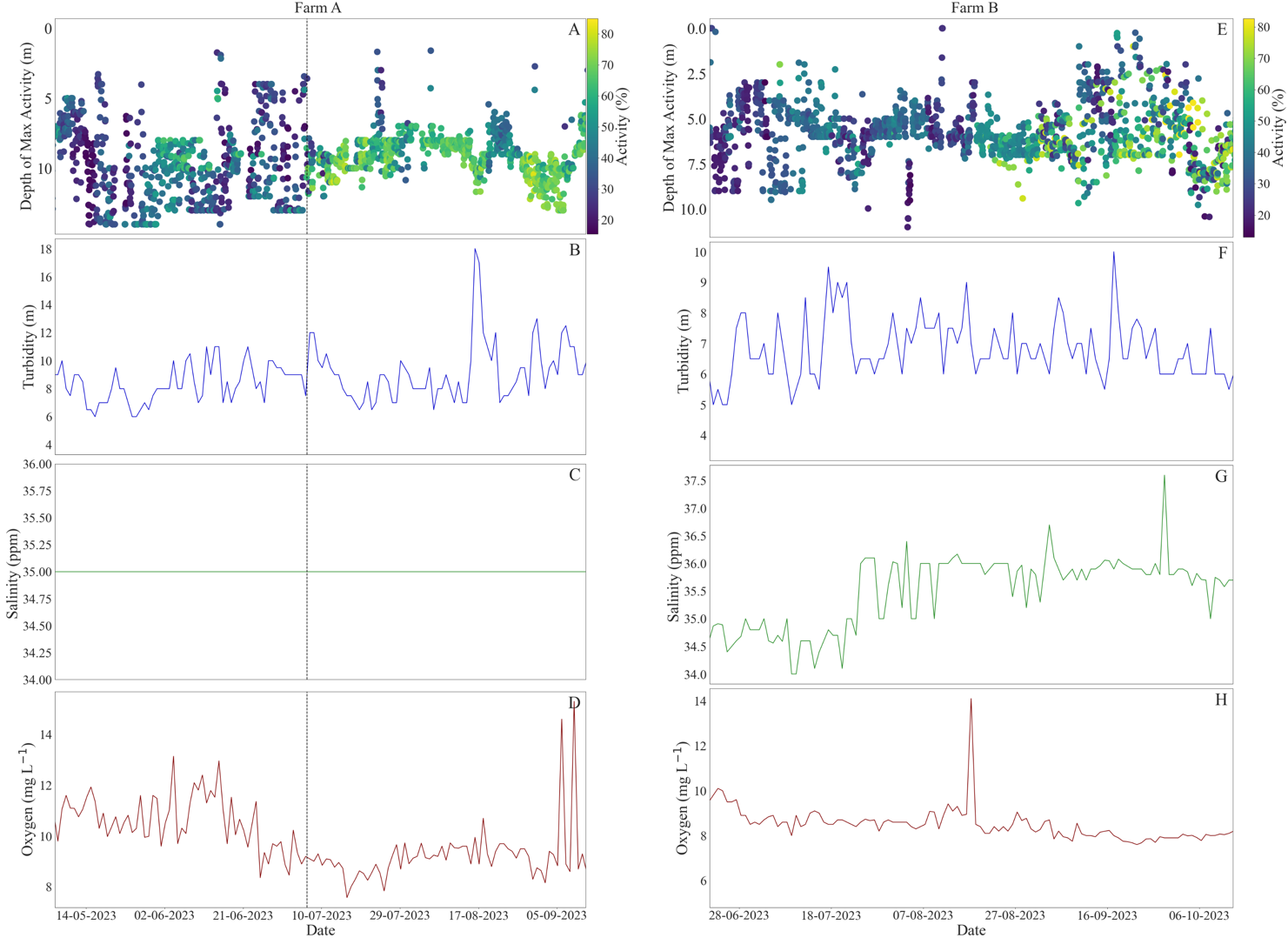
*

Figure A.3 Water quality parameters measured daily throughout the study period from the barge visible in Fig. 1 at each site. Panels show the depth of maximum activity with colours indicating the hourly-averaged activity levels observed at that depth (a,e), turbidity (m) measured with a Secchi disk (b, f), salinity (ppm), and dissolved oxygen (mg L^-1^). Note: given the consistent reading of salinity at Farm A, this suggests it was an approximation rather than precisely measured.

Table A.1 Mean and median errors between the AI algorithm and human labellers

| **Metric** | **Activity** |
| --- | --- |
| Mean Error | -2.6% |
| Mean Average Error | 9.81% |
| Median Error | 7% |
